# Influence of patient race on administration of analgesia by student paramedics

**DOI:** 10.1186/s12873-019-0245-2

**Published:** 2019-05-06

**Authors:** Bill Lord, Sahaj Khalsa

**Affiliations:** 1 School of Nursing, Midwifery and Paramedicine, University of the Sunshine Coast, 90 Sippy Downs Drive, Sippy Downs, QLD 4556 Australia; 2 Emergency Medical Services Institute & AHAC, Santa Fe Community College, 6401 Richards Ave, Santa Fe, NM 87508 USA

**Keywords:** Paramedic, Emergency care, Prehospital, Pain management, Race, Analgesia

## Abstract

**Background:**

Disparities in the management of pain are associated with factors that include social status, age and race. As there is limited data regarding the influence of race on analgesia provided by paramedics this study investigated associations between patient race and student paramedic management of pain.

**Methods:**

Retrospective study of student paramedic records entered in the FISDAP Skill Tracker database between 1 January 2014 to 31 December 2015. Cases were extracted if aged 16 to 100 years, the patient was alert and the primary or secondary impression was trauma. The primary outcome of interest was the association between patient race and student paramedic administration of any analgesia. The adjusted odds of patients receiving any analgesic was tested with logistic regression using a stepped modelling approach.

**Results:**

59,915 cases were available for analysis; median age was 50 years (IQR 39 years), 50.1% were female (*n* = 30,040). Fall was the most common case type 43% (*n* = 26,009) of cases. 14.1% of patients received any analgesia (*n* = 8424). Caucasian patients have significantly higher odds of receiving analgesia than non-Caucasian patients (*p* < 0.001). When analgesic administration is adjusted for gender, age category and injury cause, African Americans have the lowest logged odds of receiving any analgesia when compared to Caucasian patients (OR 0.60, *p* < 0.001).

**Conclusion:**

The results indicate inequality in the provision of analgesia by student paramedics based on patient race. This suggests a need for interventions to reduce disparities in care based on race.

## Background

Disparities in the provision of health care have been described in the literature, and these include disparities associated with age and gender as well as race or ethnicity. A landmark report on racial and ethnic disparities in health care from the US Institute of Medicine found that African American individuals receive fewer procedures and poorer-quality medical care than white individuals [1]. The cause of racial disparities is complex, and encompasses patient, provider and health system issues. Provider factors include prejudice and stereotyping based on patient characteristics that may influence clinical judgements and treatment decisions [2]. Disparities in the management of pain have been shown to be associated with race, with a study of the prescribing of opioids in the emergency department finding that white patients reporting pain were more likely to receive an opioid than Black, Hispanic, or Asian/other patients [3]. Evidence from other treatment settings has revealed racial and ethnic disparities in acute, chronic and palliative pain care across the lifespan, and this has led to calls for reform of health care agendas and public health policy to reduce and eliminate disparities in care [4, 5].

Pain is a commonly encountered complaint in paramedic practice, and despite having guidelines for the assessment and management of pain and effective analgesics to manage this symptom, inadequate analgesia has been frequently reported, with paramedic attitudes identified as a potential barrier to appropriate care due to the effect of affective bias on clinical judgements [6]. Despite the evidence of racial disparities arising from other health settings, there is limited research describing associations between patient race and the paramedic provision of analgesia. This study therefore aims to examine associations between patient race and paramedic student records of pain management completed during field internships where the veracity of the record was confirmed by the supervising paramedic.

## Methods

### Study design

This retrospective study of de-identified patient care data generated by student paramedics for the fulfillment of assessment requirements was governed by an existing Institutional Review Board approval from Inver Hills Community College, Inver Grove Heights, Minnesota.

### Ethics approval and consent to participate

Approval to use this existing collection of non-identifiable human data for this study was granted by the Human Research Ethics Committee of the University of the Sunshine Coast, approval number E/16/058.

### Study setting and population

Data for this study was extracted from the database for a web-based learning management system (FISDAP Skills Tracker, Inc., Minneapolis) used to record student management of cases attended during the field experience component of the paramedic training program. This system is used across most states in the United States of America. Paramedic students use this system to self-report their management of each patient encounter during the field internship. The information recorded includes patient age, gender and race as well as chief complaint, primary impression, mechanism of injury, pain severity score, vital signs and treatment. Students in this setting deliver patient care within a scope of practice that enables the development of competencies that include the administration of parenteral analgesics for the management of pain [7]. This student database was selected as it enables analysis of a large dataset of patient care provided by students under direct supervision. Other national datasets of case records that include paramedic treatment such as the National Emergency Medical Services Information System (NEMSIS) lack reliable data fields required to study variables that may be associated with analgesic administration [8].

### Study protocol

This retrospective cohort study used a convenience dataset of all student paramedic records generated between 1 January 2014 to 31 December 2015 that met the following inclusion criteria:

- Age greater than 15 years and less than or equal to 100 years;
- Alert and orientated = Yes; and
- Primary or Secondary impression = trauma (Abdominal, Chest, Extremity, Neck-back, Multi) OR burns.

Only records generated by paramedic students were extracted, as lower levels of Emergency Medical Technician (EMT) have a scope of practice that restricts the administration of certain analgesics. Students must have consented to the deidentified use of the data for research, with consent sought at the time of establishing a new student account to access the system. Data was restricted to cases where the paramedic supervisor had verified the accuracy of the student record. Head injury was an exclusion criterion as some Emergency Medical Service (EMS) treatment protocols list head injury as a contraindication to analgesia. As a record of pain severity using a validated pain severity scale is known to be infrequently captured by paramedics in the US, trauma was used as a surrogate for the potential for pain [9]. Students recorded administration of analgesia using the following predetermined categories: opioid (morphine sulfate, fentanyl, hydromorphone, meperidine), nonsteroidal anti-inflammatory drug (ketorolac, ibuprofen), or acetaminophen. Race could be recorded using the following pre-determined categories: African American, Asian, Hispanic, Native American, Caucasian or other, which are consistent with the US Census Bureau classifications of race [10]. Where pain severity was recorded, a value between zero and 10 was derived from the use of a Verbal Numeric Rating Scale where zero represents no pain and 10 the worst pain imaginable. Pain severity was categorized as mild [1–4], moderate [5–7] and severe [8–10].

### Outcomes of interest

The primary outcome of interest was the association between patient race and student paramedic administration of any analgesia for cases meeting inclusion criteria. Secondary outcomes of interest were the effect of age and gender on analgesia administration. The administration of any analgesic was presumed to be verified by the clinical preceptor. Associations between the race of the student or preceptor and analgesia administration were not included as this data was not reliably recorded.

### Data analysis

Descriptive statistical tests were used to analyze the sample population, with one-way tables presenting patient age, race, ethnicity, pain score, analgesia administration, and type of analgesia administered.

Pearson’s chi-square test was used to test for associations between the outcome of interest and predictor variables. The adjusted odds of patients receiving any analgesic was tested with logistic regression using a stepped modelling approach, with variables progressively added to the original model to assess any change in the odds of receiving any analgesia by race. Variables were included in the binomial logistic regression if they were significantly associated with analgesia administration (*p* < 0.05), or if they were expected to be an important explanatory variable based on the literature. All explanatory variables presented in Table 1, except gender (*p* > 0.05) were found to be significantly associated with analgesia administration (*p* < 0.05).

**Table 1:** Patient characteristics stratified by analgesic administration (any)

Variable	All patients	Analgesic	No analgesic	*P*-value
Number of patients, n (row %)	59,915	8424 (14.06)	51,491 (85.94)	
Age, median (IQR)	50 (39)			
Sex				
Male, n (col %)	29,780 (49.70)	4168 (49.51)	25,612 (49.83)	0.593
Female, n (col %)	30,040 (50.14)	4250 (50.49)	25,790 (50.17)	
Missing, n (col %)	95 (0.16)			
Initial pain severity score, median (IQR)	7 (4)			
Ethnicity				0.000
African American, n (col %)	6385 (10.66)	662 (11.27)	5723 (16.08)	
Asian, n (col %)	597 (1)	68 (1.16)	529 (1.49)	
Caucasian, n (col %)	29,281 (48.87)	4449 (75.73)	24,832 (69.79)	
Hispanic, n (col %)	3959 (6.61)	534 (9.09)	3425 (9.79)	
Native American, n (col %)	311 (0.52)	39 (0.66)	272 (0.76)	
Other, n (col %)	538 (0.90)	68 (1.16)	470 (1.32)	
Unspecified, n (col %)	385 (0.64)	55 (0.94)	330 (0.93)	
Missing, n (col %)	18,459 (30.81)			
Pain score category				0.000
Mild, n (col %)	8875 (14.8)	495 (10)	8380 (39.3)	
Moderate, n (col %)	11,563 (19.3)	2282 (46.2)	9281 (43.5)	
Severe, n (col %)	5834 (9.7)	2164 (43.8)	3670 (17.2)	
Missing, n (col %)	33,643 (56.2)			
Age category (years)				0.000
16–40, n (col %)	23,408 (39.07)	2972 (35.28)	20,436 (39.69)	
41–60, n (col %)	15,646 (26.11)	2339 (27.77)	13,307 (25.84)	
61–80, n (col %)	13,035 (21.76)	1966 (23.34)	11,069 (21.50)	
81–100, n (col %)	7826 (13.06)	1147 (13.62)	6679 (12.97)	
Cause of injury				0.000
Fall, n (col %)	26,009 (43.41)	4672 (64.24)	21,337 (47.91)	
Family violence, n (col %)	1339 (2.23)	81 (1.11)	1258 (2.82)	
Firearm, n (col %)	909 (1.52)	133 (1.83)	776 (1.74)	
Stabbing/cutting, n (col %)	2718 (4.54)	147 (2.02)	2571 (5.77)	
Environmental, n (col %)	273 (0.46)	33 (0.45)	240 (0.54)	
Transport, n (col %)	17,629 (29.42)	1755 (24.13)	15,874 (35.65)	
Toxicological, n (col %)	119 (0.2)	7 (0.1)	112 (0.25)	
Trauma other, n (col %)	2810 (4.69)	445 (6.11)	2365 (5.31)	
Missing, n (col %)	8109 (13.53)			

Stata version 14 (Stata Corporation, College Station, Texas) was used to undertake the statistical analysis. Stata uses listwise deletion of missing observations. As a stepwise forward-inclusion regression approach was chosen for the analysis, the analytic sample needed to be established first, otherwise, it was possible new observations would be added into the analysis with each new variable added, resulting in a different n for each model. Further, any comparison of the R-squared between each model with different observations in each model would be redundant. In order to establish the analytic sample for the binomial logistic regression analysis, a model with all independent variables included was first calculated; all subsequent models used in the analysis (Models 1–5) used only the observations saved in the analytic sample (*n* = 17,729) using the Stata e(sample) function. A forward-inclusion approach was chosen so changes in the significance level of the race variables could be observed. A forward inclusion stepwise approach also allows changes in the R-squared to be observed.

The first model included only dichotomous white or other race categories. The base (comparison) category is white patients. The second model added patient gender with male as the base category. The third model added in patient age with the 18–40 category as the base variable. The fourth models added patient pain score category with moderate pain as the base category. Lastly, in the fifth model, cause of injury was added with fall (as the category with the most observations) as the base category.

The same approach was repeated in a second analysis using each of the recorded race classifications to expand the non-white category and allow further understanding of the relationship between analgesia administration and race. Caucasian was used as the base category for this analysis. The post-estimation test likelihood ratios were calculated to assess for a significant difference of the nested model (smaller model) in the full model, which included all variables.

## Results

The original dataset comprised 290,670 unique cases. After applying inclusion criteria, 59,915 cases were available for analysis. Race was recorded in 41,071 of these cases, with 31.1% of cases missing race data. Pain severity was recorded in 26,272 cases (43.8%). Median age was 50 years (IQR 39 years), with 50.1% female (*n* = 30,040). The analytic sample used in the logistic regression models discussed below and presented in Table 2 comprised 17,729 unique cases.

**Table 2:** Logistic regression analysis of the odds of a patient receiving any analgesic

	AOR (95% CI)
Race	
Caucasian	1
African American	0.60 (0.53–0.69)***
Asian	0.79 (0.55–1.15)
Hispanic	0.81 (0.70–0.93)**
Native American	1.08 (0.62–1.87)
Other	0.88 (0.61–1.27)
Sex	
Male	1
Female	0.9 (0.83–0.98)*
Age (y)	
16–40	1
41–60	0.94 (0.85–1.05)
61–80	0.99 (0.88–1.11)
81–100	0.93 (0.80–1.07)
Pain severity	
Moderate	1
Mild	0.24 (0.21–0.27)***
Severe	2.39 (2.19–2.61)***
Injury cause	
Fall	1
Family violence	0.35 (0.25–0.49)***
Firearm	0.66 (0.48–0.90)**
Stabbing/cutting	0.37 (0.28–0.49)***
Environmental	1.07 (0.57–1.98)
Transport	0.58 (0.52–0.64)***
Toxicological	0.40 (0.12–1.39)
Trauma other	1.11 (0.93–1.33)

* *p* < 0.05, **p < 0.01, *** *p* < 0.001

observations 17,729

Pseudo R-squared 0.116

The most common cause of injury was falls. The cause of injury variable included 23 separate categories, and these were collapsed into 8 categories for the analysis. Categories combined included: drowning, lightning, radiation exposure, excessive cold and excessive heat into an ‘environmental’ category. Aircraft related accident, water transport accident, bicycle accident and motor vehicle accident were combined into a ‘transport’ category. A toxicological category included drug poisoning, chemical poisoning, chemical poisoning and venomous stings. Lastly, a ‘trauma other’ category was generated comprising mechanical suffocation, electrocution (non-lightning), fire or flames, rape, bites, machinery accident and smoke inhalation. Patient characteristics are shown in Table 1.

The unadjusted odds ratio for African American patients receiving analgesia is statistically significantly (*p* < 0.001) at 0.62 times that of Caucasian patients. The adjusted odds ratio for Hispanic patients receiving analgesia is statistically significant (*p* < 0.01) at 0.81 times that of Caucasian patients. The odds ratios for other racial categories included in the model (Asian, Native American and Other) were not statistically significant (*p* > 0.05). The Pseudo R-squared statistic suggests that 0.4% of variance in the outcome variable is explained by this model.

In model two, gender is added. The odds ratio for female, however, is not statistically significant (p > 0.05), suggesting that gender does not account for analgesia administration.

In model three, when patient age is added with the reference age category 18–40 the patients aged 61–80 have an odds of receiving analgesia 1.14 times that of patients age 18–40 years. The odds ratio of the African American category remains highly statistically significant, though increases slightly to an odds of 0.64. The Pseudo R-squared increases to 0.5% of variance explained in the model.

Model four adds pain score category to the analysis. Patients in severe pain had a statistically significant odds of receiving analgesia 2.45 times that of patients in moderate pain. This suggests that patients in severe pain have an increased odds of receiving analgesia in comparison to those in moderate pain. The Pseudo R-squared increased in this model to 0.104, indicating that this model explain 10.4% of variance in the outcome of receiving analgesia.

Model five adds cause of injury. Cause categories of family violence, firearm, stabbing/cutting and transport were all highly statistically significant (*p* < 0.05). The odds of an African American patient receiving an analgesic, with cause of injury included in the model, increases slightly from model four, but it is still lower than model one, suggesting that even when gender, age, pain score and cause of injury are accounted for, African American patients still have lower odds that Caucasian patients of receiving analgesia. The same conclusion can be applied to Hispanic patients as well, though they have a higher odds of receiving analgesia than African American patients. Table 2 presents a logistic regression analysis, including all independent variables, of the odds of a patient receiving any analgesic.

## Discussion

Previous studies have shown disparities in patient management for minority patients. These results have held true in multiple disciplines, but almost all studies have examined patients who are in a healthcare facility, not in the prehospital environment. Previous prehospital research has described an association between patient race and paramedic management of suspected cardiac chest pain. However, that study was limited by poor reporting of race and a very small number of non-white patients in the dataset [11]. One recent study reported a low frequency of paramedic administration of analgesics for painful injury and a reduced frequency of analgesia for Black patients. However, this study did not control for variables that may be associated with a reduced odds of analgesia administration [8].

This study contributes to the understanding of disparities in EMS by describing the association between race and analgesia administration by paramedic students, and reaffirms the pattern previously reported of suboptimal care being provided to minority patients.

How racial disparities are mitigated is a complicated question. Disparities affecting pain management decisions may arise from complex interactions between patient race, racial bias of the care provider, context and clinical ambiguity [12]. Professional associations and educational programs that train EMTs and paramedics should increase their conversations around healthcare disparities. Education should include guided reflective strategies to help the student understand pain from the patient’s perspective, as these strategies have been shown to induce empathy and reduce racial bias associated with the management of pain [13]. EMS training programs should ensure that educational design aims to increase their students’ exposure to multi-racial patients in all settings and across the lifespan. Introducing minority patients during simulation, using cognitive debriefing to carefully evaluate differences in treatment from similar cases involving white patients, and teaching that helps students understand the effect that subconscious bias often has on the quality of care may help to address disparities based on race. Currently, many EMS training manikins have ‘white’ skin, which means students may not have opportunities to treat non-white patients. As manufacturers now supply different skin tones for their training manikins, training programs should make concerted efforts to purchase manikins that better represent the patients their students will ultimately treat. At a national level, EMT education standards need to address the lack of detail regarding the development of cultural competency, as current standards fail to adequately address this need [7].

While education influences the practice of new graduates, these disparities are unlikely to be addressed solely through education. If EMT and Paramedic graduates begin work in a system which fails to asses for and mitigate racially based disparities in treatment, those graduates may inevitably conform to system norms. It is critically important, therefore, for those systems to actively assess for such disparities and design interventions that aim to reduce disparities [14]. EMS and Fire Department Quality Assurance and Quality Improvement processes (QA/QI) are typically designed to evaluate compliance with treatment protocols, but this process may not have the capacity to identify disparities in the management of specific case types across their system. Previous research has shown that clinician behavior can change when that behavior is specifically measured and reported [15]. QA/QI programs must consistently look for, measure and evaluate disparities such as those associated with race. Without robust investigations for racially based disparities in treatment, those disparities will likely remain.

This study also found a low (14%) rate of analgesia administered to patients with a complaint of pain. While informed patient refusal of analgesia and clinical guideline or protocol constraints may account for some of this finding, the low frequency of analgesia administration replicates previous research and requires further study and actions to improve pain management in this setting [8]. Patient disposition or lack of expected behavioral response to pain may be associated with cultural differences. Some EMS providers may believe that a patient not requesting pain management means that they do not require analgesia. If true, this belief is contrary to our responsibility to advocate on behalf of patients and to provide advice regarding the risks and benefits of treatment options.

### Limitations

The means of classifying race in this study cannot be determined. As such paramedic students may have classified the patient based on appearance rather than asking the patient to self-report their race. This may have caused inaccurate recording of race. Only one racial category can be selected by the student, and this prevents identification of patients who may identify as multi-racial. In addition, there was considerable missing data regarding race.

The rate of patient refusal to consent to analgesia cannot be determined from the data. Hence, rates of refusal to consent to treatment may have influenced the outcome.

The influence of the paramedic preceptor on student paramedic clinical decisions may have been a factor that influenced results. As such a further large study of qualified paramedic practice is warranted.

Errors in the documentation of care cannot be identified in this retrospective study of student paramedic case management. The data represents a record of patient care that is a requirement of the student’s enrolment in a paramedic training program, and as such may not achieve the same level of reliability as a patient care record generated to document care and enable clinical audit. However, only records that were marked as checked by the preceptor were included in this study to offset this potential for error.

## Conclusion

Caucasian patients have significantly higher odds of receiving analgesia from a student paramedic in this study than non-Caucasian patients. When ethnicity is considered more closely, it is found that African Americans have the lowest odds of receiving any analgesia when compared to Caucasian patients. This adds to the evidence for disparities in the management of pain based on patient race and reinforces the need for clinical audit by EMS to identify and mitigate disparities in the quality of care based on patient characteristics such as race. Evidence arising from studies that aim to change health provider behavior to ensure equitable delivery of care should inform EMT education programs, in-service quality improvement education, and system design to eliminate racial disparities associated with pain management.

## Abbreviations

EMS: Emergency Medical Service
EMT: Emergency Medical Technician
NEMSIS: National Emergency Medical Services Information System
QA/QI: Quality Assurance and Quality Improvement
